# Autosomal dominant hereditary spastic paraplegia: Novel mutations in the *REEP1 *gene (SPG31)

**DOI:** 10.1186/1471-2350-9-71

**Published:** 2008-07-21

**Authors:** Katharina J Schlang, Larissa Arning, Joerg T Epplen, Susanne Stemmler

**Affiliations:** 1 Department of Human Genetics, Ruhr-University, 44801 Bochum, Germany

## Abstract

**Background:**

Mutations in the *SPG4 *gene (spastin) and in the *SPG3A *gene (atlastin) account for the majority of 'pure' autosomal dominant form of hereditary spastic paraplegia (HSP). Recently, mutations in the *REEP1 *gene were identified to cause autosomal dominant HSP type SPG31. The purpose of this study was to determine the prevalence of *REEP1 *mutations in a cohort of 162 unrelated Caucasian index patients with 'pure' HSP and a positive family history (at least two persons per family presented symptoms).

**Methods:**

162 patients were screened for mutations by, both, DHPLC and direct sequencing.

**Results:**

Ten mutations were identified in the *REEP1 *gene, these included eight novel mutations comprising small insertions/deletions causing frame shifts and subsequently premature stop codons, one nonsense mutation and one splice site mutation as well as two missense mutations. Both missense mutations and the splice site mutation were not identified in 170 control subjects.

**Conclusion:**

In our HSP cohort we found pathogenic mutations in 4.3% of cases with autosomal dominant inheritance. Our results confirm the previously observed mutation range of 3% to 6.5%, respectively, and they widen the spectrum of *REEP1 *mutations.

## Background

Hereditary spastic paraplegia (HSP) is a clinically and genetically heterogeneous disorder characterised by progressive spasticity, weakness and hyperreflexia of the lower limbs. Clinically it is divided into a 'pure' or uncomplicated form with progressive spasticity as major symptom, associated with brisk reflexes, muscle weakness, positive Babinski's sign and urinary urgency as well as a 'complicated' form accompanied with other neurological abnormalities, i.e. retinal pigmentation, mental retardation, deafness, optic atrophy or seizures [1,2]. So far, over 35 loci have been identified for autosomal dominant, recessive and X-linked HSP. The autosomal dominant HSP is indeed the main mode of inheritance, accounting for about 70–80% of all HSPs in Western countries, and it is predominantly associated with pure forms [1]. The occurrence of recessive HSP amount to 20–30%, whereas X-linked HSP is very rare.

In 15–40% of families with the 'pure' autosomal dominant form of HSP, DNA sequence analysis identified a mutation in the *SPG4 *gene (spastin) [1] and in ~10% a mutation in the *SPG3A *gene (atlastin) [2]. In further 18–20% of families gross deletions (of one or more exons) of the *SPG4 *gene have been found [3,4]. No pathogenic gross deletions in the *SPG3A *gene have been demonstrated so far. Mutations in the *REEP1 *gene (SPG31) were analysed in two studies [5,6], and pathogenic mutations were identified in 6.5% and 3% of cases, respectively, including one large multi-exonic duplication [5,6].

## Methods

### Subjects

162 unrelated patients with clinical symptoms of 'pure' HSP and a positive family history (at least two persons per family presented symptoms) were analysed in this study. Mutations in the *SPG4 *gene were excluded in all patients and in *SPG3A *in 152 of the 162 patients. Gross deletions were excluded in half of the patients by MLPA. Control samples from 170 healthy German blood donors were collected at the Department of transfusion medicine of the University Hospital in Essen (Germany). DNA was extracted from EDTA-anticoagulated peripheral blood by using a standard salting-out method [7]. This study was approved by the institutional review board, informed consent was obtained from all subjects, and the Declaration of Helsinki protocols were followed.

### PCR

Screening of the entire coding region of the *REEP1 *gene (seven exons including the flanking intronic sequence, at least 40 bp from each end) was performed using polymerase chain reaction (PCR) with consecutive DHPLC analysis. PCR reactions for exon 1 were performed in a total volume of 10 μl, containing 60 ng DNA, 10 mmol of each dNTP DEAZA (Roche, Mannheim, Germany), 3 mmol MgCl_2 _(Qiagen, Hilden, Germany) 10 pmol of each primer, 0.25 U Taq polymerase Hotstar Taq Plus (Qiagen, Hilden, Germany), DMSO 5% and 10 × buffer (contains 15 mM MgCl_2_); (Qiagen, Hilden, Germany). PCR reactions for exons 2 to 7 were performed in a total volume of 12.5 μl, containing 100 ng DNA, 2.5 mmol of each dNTP, 2 mmol MgCl_2_, 10 pmol of each primer, 0.5 U Taq polymerase (Genecraft, Lüdinghausen, Germany) and 10 × buffer BioTherm without MgCl_2 _(Genecraft, Lüdinghausen, Germany). Thermal cycling was performed in a Biometra TGradient (Whatman, Maidstone, Kent, England). For each of the 7 PCR fragments, primer sequences and annealing temperatures are shown in Table 1.

**Table 1:** Primer sequences and annealing temperatures

Amplified segment	Primer	Annealing temperature
*SPG31 *Ex 1	F1: ACCAGCTCACCGCCCAATC R1: GTATTAATAGCCCGAGCCACTGG	56°C/30 cycles
*SPG31 *Ex 2	F2: AAGATAGAGGTGCCCAAGGATG R2: TGAGAACTGTGATGAAGGAACTCC	58°C/28 cycles
*SPG31 *Ex 3	F3: GGAAGGATAGGGAGAAGGCTAC R3: CCTGAGGTGAGGTTTGAGGC	58°C/28 cycles
*SPG31 *Ex 4	F4: TTCAAAAGGGCAGTGTTGCTG R4: ATGACTTGCTGGAGGCAAATGA	58°C/28 cycles
*SPG31 *Ex 5	F5: GCCAAGCCCAATGCTCAGAG R5: GGTCCTTAGCCTGTTCTGTGTGG	60°C/28 cycles
*SPG31 *Ex 6	F6: AGCTCCCTCTTGGCCTTTGTC R6: GGACTGGGCCTCTCTCAATGA	58°C/28 cycles
*SPG31 *Ex 7	F7: GAGATTCAGGGAGACCCCAGC R7: GGAGAGAGAAAAGGCCATGTTTG	60°C/28 cycles

(F: forward primer; R: reverse primer)

### DHPLC analyses

DHPLC is a chromatographic mutation analysis technique that is based on temperature-dependent separation of DNA from PCR-amplified DNA fragments [8]. Different schemes for mutation screening were performed using DHPLC, i.e. the WAVE^® ^DNA Fragment Analysis System (Transgenomic, Glasgow, UK) as described [9] with the analysis software Navigator Software (Transgenomic, Glasgow, UK).

PCR mixes were applied in double and four-fold amount (conditions see above) and were performed in total volumes of 25 μl (exons 2 to 7) or 40 μl (exon 1). Additionally the PCR amplicons were heated up to 94°C for 3 min and cooled slowly down to 20°C (30 sec) in a thermocycler (Biometra TGradient, Whatman, Maidstone, Kent, England) before loading onto the WAVE column. The amount of one injection per PCR sample was 5 μl. The probes were analyzed with variable retention times (Table 2) and compared to a chromatogram of a healthy control sequence variation. A variant in exon 3 (c.164C>A, p.Thr55Lys) was genotyped using the WAVE analysis system (no restriction enzyme available).

**Table 2:** WAVE conditions

Amplified segment	WAVE: oven temperature	time shift
*SPG31 *exon 1	65°C/70°C	no
*SPG31 *exon 2	54,5°C/57°C/58°C	no
*SPG31 *exon 3	59°C/63°C	63°C: 2,0
*SPG31 *exon 4	56°C/57°C/59°C	59°C: 1,0
*SPG31 *exon 5	58°C/61,5°C/64°C	no
*SPG31 *exon 6	61°C/62°C/63°C	no
*SPG31 *exon 7	58,6°C/60,6°C	no

### DNA sequencing

DNA samples with peculiar separation behaviour in DHPLC analysis were directly sequenced using the AMPure- (1^st ^purification) and CleanSeq-Kit (2^nd^-purification; Beckman, Krefeld, Germany) as described in the manufacturer's instructions on an automated sequencer (MegaBace 1000, GE Healthcare, Freiburg, Germany; and ABI PRISM 377, DNA Sequencer).

### RFLP

Two newly identified sequence variations, c.320T>C, p.Leu107Pro (exon 5) and c.105+6T>C (intron 2), as well as a mutation in the 3'UTR (c.606+43G>T) were genotyped by restriction enzyme digestion. Patient and control samples were amplified using the same primers and conditions as described above. 12.5 μl of PCR products were digested with 0.31 units of Bbv I (intron 2), 0.25 units of Msp I (exon 5) and 0.46 units of BsuRI (3'UTR) at 37°C for 3 hours. The fragments were separated on 2% agarose gels in 1 × TBE buffer (30 min, 200 V) and visualised using ethidium bromide.

### *In silico *analysis

Putative functional and splicing effects of the three unclear sequence variations were investigated using the programs ESEfinder release 3.0 [10] and NNSPLICE 0.9 software [11]. ESEfinder identifies putative exon splicing enhancers responsive to the human SR proteins SF2/ASF, SC35, SRp40 and SRp55. NNSPLICE is based on a neural network to predict the presence and relative efficiencies of donor and acceptor sites.

## Results and Discussion

In this study we analysed a cohort of 162 patients with the 'pure' autosomal-dominant form of HSP for mutations in the *REEP1 *gene. In addition, 170 healthy control subjects were analysed for the newly identified sequence variations.

We identified nucleotide exchanges in all exons in comparison to the reference sequence except for exon 1. Ten mutations which include 7 (4.3% of cases) pathogenic mutations (small insertions/deletions, nonsense- and 3'UTR exchanges) and three (1.9% of cases) novel sequence variations (missense and splice site) were identified. Six of these seven pathogenic mutations (three small out of frame deletions, two small out of frame insertion and a nonsense mutation) as well as three unclear sequence variations (two missense and one splice site) have not been described before (Table 3). In detail, we newly identified two small deletions of one nucleotide (c.60delG and c.478delA), one deletion of eight nucleotides (c.340_347delAGTTACGA), an insertion of one nucleotide (c.419_420insG), an insertion of two nucleotides (c.183_184insCT) and a nucleotide exchange which leads to a premature stop codon (c.C345A; p.Try115X). Because DHPLC mutation screening technique is not 100% sensitive [12], sequence alterations might be missed, which would increase the frequency of *REEP1 *mutations. In three cases more than one symptomatic family member was analysed. There was one more family member tested for the deletions in exon 6 (c.478delA) and in exon 2 (c.60delG) in each case and two additional family members for the insertion (c.419_420insG). The three families are composed of father and son, father and daughter as well as mother and two children. All affected family members show the same mutation. In our HSP cohort the mean age of onset is in the early twenties (3 to 50 years). Two novel variations represent missense mutations (c.164C>A, p.Thr55Lys, c.320T>C, p.Leu107Pro) and the other one a splice site variation in the donor splice site (c.105+6 T>C). The splice site prediction program NNSPLICE 0.9 calculates a score of 0.98 for the wild-type donor of exon/intron 2. The c.105+6 T>C substitution lowers the donor consensus value to 0.88, probably leading to preferential usage of a cryptic donor present 29 bp downstream in intron 2 with a score of 0.64 [gagtcagGTcaggtg].

**Table 3:** Mutations found in *REEP1*

	Nucleotide exchange	Amino acid exchange
*SPG31 *exon 1	none	
*SPG31 *exon 2	c.60delG	p.6fsX
	c.105+6T>C	
*SPG31 *exon 3	c.164C>A	p.Thr55Lys
*SPG31 *exon 4	c.183_184insCT	p.7fsX
*SPG31 *exon 5	c.320T>C	p.Leu107Pro
	c.340_347delAGTTACGA	p.69fsX,
	c.345C>A	p.Try115X
*SPG31 *exon 6	c.419_420insG	p.45fsX
	c.478delA	p.62fsX
*SPG31 *exon 7	c.606+43G>T*	

* previously described

*In silico *analysis with the ESE-finder program revealed that c.164C>A abolishes strong exon splicing enhancer sites for SF2/ASF, SRp40 and SRp55 as well as it leads to a new strong site for SRp55. For c.320T>C the score for a potential binding site for the splicing factors SRp40 strongly increases when substituting the T by C. Thus these sequence exchanges could result in defective splicing of introns, exon skipping, frameshift and introduction of another premature termination codon. In that case, all sequence variations found would be in line with the concept of haploinsufficiency. Unfortunately, we could not test this hypothesis, because, as *REEP1 *is not expressed in peripheral blood cells [6], we were not able to perform RNA analyses. Furthermore, we also had no further relatives with symptoms of HSP available to be investigated. Segregation analysis for these sequence variations is advisable in order to confirm the pathogenetic relevance. Yet, all unclear sequence variations were absent in 170 healthy controls.

In addition, we identified also common polymorphisms in exon 2 to exon 5 in many cases, which are listed in Table 4. Additionally, we found two further polymorphisms in intron 6 (c.595T>G) and the 3'UTR (c.606+155T>C) in four of the 162 patients in heterozygous state (allele frequency 1.2%).

**Table 4:** Polymorphisms found in *REEP1*

	rs-number/Polymorphism
*SPG31 *intron 1	rs1863059
*SPG31 *intron 2	rs1863058
	rs1863056
*SPG31 *exon 4	rs2276625
*SPG31 *intron 5	rs12988844
*SPG31 *intron 6	c.595-19T>G, G allele: 1,2%
*SPG31 *3'UTR	c.606+155T>C, C allele: 1,2%

## Conclusion

Having identified pathogenic mutations in the *REEP1 *gene in 4.3% of our HSP cases, previous mutation frequency determinations (6.5% and 3%, respectively) are substantiated, and hence the spectrum of *REEP1 *mutations is complemented. Mutations in the *REEP1 *gene appear as the third most frequent cause in families with the 'pure' autosomal dominant form of HSP. Therefore mutation analysis in the *REEP1 *gene should be included in the comprehensive care for patients with the 'pure' autosomal dominant form of HSP.

## Abbreviations

HSP: hereditary spastic paraplegia; REEP1: receptor expression enhancing protein 1; 3'UTR: 3'untranslated region; PCR: polymerase chain reaction; DHPLC: denaturating high-performance liquid chromatography; DEAZA: 7-deaza-2'-deoxyguanosine; DMSO: dimethyl sulphoxide; RFLP: restriction fragment length polymorphism; MLPA: multiplex ligation dependent probe amplification.

## Competing interests

The authors declare that they have no competing interests.

## Authors' contributions

KS performed the experiments and drafted the manuscript. SS, LA and JTE participated in the design and coordination of the study and finalised the manuscript. All authors read and approved the final version of the manuscript.

## Pre-publication history

The pre-publication history for this paper can be accessed here:


